# Abnormal Spontaneous Brain Activity in Early Parkinson’s Disease With Mild Cognitive Impairment: A Resting-State fMRI Study

**DOI:** 10.3389/fphys.2018.01093

**Published:** 2018-08-14

**Authors:** Zhijiang Wang, Xiuqin Jia, Huimin Chen, Tao Feng, Huali Wang

**Affiliations:** ^1^Peking University Institute of Mental Health (Sixth Hospital), Beijing, China; ^2^National Clinical Research Center for Mental Disorders and Key Laboratory of Mental Health, Ministry of Health, Peking University, Beijing, China; ^3^Beijing Municipal Key Laboratory for Translational Research on Diagnosis and Treatment of Dementia, Beijing, China; ^4^Department of Radiology, Beijing Chaoyang Hospital, Capital Medical University, Beijing, China; ^5^Center for Neurodegenerative Disease, Department of Neurology, Beijing Tiantan Hospital, Capital Medical University, Beijing, China; ^6^China National Clinical Research Center for Neurological Diseases, Beijing, China

**Keywords:** Parkinson’s disease, mild cognitive impairment, resting-state fMRI, amplitude of low-frequency fluctuations (ALFF), right inferior frontal gyrus

## Abstract

Mild cognitive impairment (MCI) is a common symptom at the baseline of early Parkinson’s disease (PD) diagnosis, but the neural mechanism is unclear. To address the issue, the present study employed resting-state functional magnetic resonance imaging data of 19 drug-naïve PD patients with normal cognition (PD-NC), 10 PD patients with MCI (PD-MCI) and 13 age- and gender-matched healthy controls (HC) from the Parkinson’s progression markers initiative (PPMI) (http://www.ppmi-info.org/), and examined abnormal spontaneous brain activities in the PD-MCI. The pattern of spontaneous brain activity was measured by examining the amplitude of low-frequency fluctuations (ALFF) of blood oxygen level dependent signal. Voxel-wise one-way analysis of covariance and *post hoc* analyses of ALFF were performed under non-parametric permutation tests in a general linear model among the three groups, with age, gender and data center as additional covariates. Statistical significances in the *post hoc* analysis were corrected by a small volume correction with a cluster-level threshold of *p* < 0.05 (*n* = 10000 permutations, FWE-corrected). Correlations of clinical and neuropsychological assessments [i.e., Unified Parkinson’s Disease Rating Scale (UPDRS) total score, Montreal Cognitive Assessment (MoCA) and cognitive domains] with the regional ALFF were performed in the PD-MCI group. Compared with the HC, both PD groups exhibited reduced ALFF in the occipital area (Calcarine_R/Cuneus_R). Specially, the PD-MCI group additionally exhibited increased ALFF in the opercular part of right inferior frontal gyrus (Frontal_Inf_Oper_R). Comparing with the PD-NC, the PD-MCI group exhibited significantly higher ALFF in the Frontal_Inf_Oper_R and left fusiform gyus (*ps* < 0.05). The correlation analysis revealed that the ALFF in the Frontal_Inf_Oper_R was positively correlated with the UPDRS total score (*p* < 0.05), but marginally negatively correlated with the MoCA score. For cognitive domains, the ALFF in the region also showed a significantly negative correlation with the score of SF test (*p* < 0.01) and a marginally negative correlation with the score of Symbol-Digit Modalities Test. Together, we concluded hyperactivity in the right inferior frontal gyrus in early PD with MCI, suggesting a compensatory recruitment in response to cognitive decline, which may shed light on thought of dementia progression and potentially comprehensive treatment in PD.

## Introduction

Parkinson’s disease (PD) is a progressive neurodegenerative disorder characterized primarily by motor symptoms of bradykinesia, tremor, rigidity, and gait/postural disturbance. However, growing evidence suggests it may commonly lead to many non-motor psychiatric symptoms as well since at the early stage of this disease, such as cognitive impairment (e.g., verbal, visuospatial, executive, and memory functions) and depression (Chaudhuri and Schapira, 2009), and dementia is a frequently final complication of the late symptoms in PD patients (Aarsland et al., 2003; Garcia-Ptacek and Kramberger, 2016). Moreover, mild cognitive impairment (MCI) has been identified as an important risk factor to dementia at the early PD diagnosis and strongly predicts the progression to PD dementia (Anang et al., 2014), since it occurs most frequently with aging and duration of PD (Litvan et al., 2012). Thus, the notion of MCI in PD is valuable of understanding PD development and associated dementia. However, it has not been particularly well understood of the neural basis underlying the MCI in PD.

Over the past decade, resting-state functional magnetic resonance imaging (R-fMRI) has been widely developed as a non-invasive and effective technology to examine spontaneous brain activity in both normal functions and neuropsychiatric disease pathophysiology (Biswal et al., 1995; Fox and Raichle, 2007). Amplitude of low-frequency fluctuations (ALFF) is a widely used measurement for the local neural activity in low-frequency (0.01-0.1 Hz) fluctuations of the blood oxygen level dependent (BOLD) signal during rest (Zang et al., 2007). It has also been effectively used to investigate abnormal spatial patterns of spontaneous brain activity in neurodegenerative diseases, including PD (Hou et al., 2014; Chen et al., 2015; Xiang et al., 2016; Pan et al., 2017; Tang et al., 2017), Alzheimer’s disease (Wang et al., 2011; Liu et al., 2014) and MCI (Han et al., 2011; Wang et al., 2011). On the one hand, studies in PD have shown widespread abnormalities of the ALFF of the whole brain spontaneous activity in a range of brain regions, such as the motor cortices, striatum, cerebellum, and brain stem, but also emphasize that these different findings depend on PD subtypes (i.e., tremor-dominant, posture instability gait difficulty, and non-motor symptoms) (Chen et al., 2015; Pan et al., 2017) and BOLD fluctuation frequency (Hou et al., 2014). On the other hand, studies in MCI have shown the whole brain ALFF values are decreased mainly in the default mode network and increased in several widespread areas including lateral temporal regions, superior frontal, occipital and parietal regions (Han et al., 2011; Wang et al., 2011). However, underlying neural correlates of cognitive impairment in early PD is unclear.

Recently, several studies have reported a range of abnormal brain areas that are associated with cognitive decline in PD. For example, R-fMRI functional connectivity analysis has suggested patients of PD with MCI could be characterized by dysfunction in the default mode network and dorsal attention network (Baggio et al., 2015; Hou et al., 2016). A longitudinal study using task-fMRI has reported reduction of BOLD signals that are related to working-memory brain activity in the right fusiform gyrus and right vermis in patients of PD with MCI (Ekman et al., 2014). Furthermore, a quite similar work with the present work applied ALFF to measure abnormalities of spontaneous brain activity in patients of PD with MCI (Gao et al., 2016). But the patients used in their study were not early PD diagnosis, without drug-naïve and depression assessment (depression is also another non-motor symptom in PD), so the results could not get rid of the influence of drug-treatment and possible mood disorder.

In the present study, we employed MRI and clinical/neuropsychological data of baseline PD patients without drug-treatment from Parkinson’s progression markers initiative (PPMI)^1^ to detect the abnormalities of brain activity that is widely associated with cognitive decline in PD. To address this issue, we used early diagnosed PD patients who do not suffer from depression and are untreated. Then, we used R-fMRI and ALFF measurement to investigate regional differences in spontaneous brain activity in the patients of PD with MCI (PD-MCI), PD with normal cognition (PD-NC) and healthy controls (HC), and also examined correlations of the ALFF values with scores of clinical and neuropsychological assessments.

## Materials and Methods

### Participants

All MRI and clinical data used in this study was downloaded from the PPMI^1^, which is a first large-scale multicenter project of PD progression biomarkers (Marek et al., 2011). According to the PPMI cohort inclusion criteria, the included baseline PD patients were required to: (i) have at least two criteria of bradykinesia, ridigity, and resting tremor or either asymmetric resting tremor or asymmetric bradykinesia; (ii) satisfy an early clinical disease stage [diagnosis of PD for less than 2 years and Hoehn and Yahr (H&Y) stage I or II]; (iii) be untreated for PD; (iv) have a dopamine transporter (DAT) deficit on imaging; (v) not have dementia as determined by the site investigators; and (vi) not suffer from depression with 15-item Geriatric Depression Scale (GDS-15) < 5 (Meara et al., 1999). Healthy controls (HC) were required to have: (i) no significant neurologic dysfunction; (ii) no first-degree family member with PD; and (iii) a Montreal Cognitive Assessment (MoCA) score greater than 26. The patients of PD with mild cognitive impairment (PD-MCI) were required to meet diagnosis of MCI defined by PD-MCI level I of the Movement Disorder Society (MDS) Task Force. The patients of PD with normal cognition (PD-NC) were required to satisfy the criteria of PD (i–v) and a MoCA greater than 26. The PPMI overall study was approved by the Research Subjects Review Board at the University of Rochester, and each site was approved by the institutional review board, and participants provided written informed consent. Considering that all participants have resting-state fMRI data and the same data-centers have both patients of PD with and without cognitive impairment, we totally acquired 19 PD-NC, 10 PD-MCI, and 13 age- and gender-matched HC participants. Data was downloaded on September 22nd, 2015 for PD and on November 25th, 2016 for HC.

These participants were totally enrolled at four PPMI sites that used a standardized protocol for three Tesla machines (all Siemens Healthcare, United States). A 3D magnetization prepared rapid gradient echo (MPRAGE) sequence was used for imaging brain anatomy (176 axial slices, repetition time = 2300 ms, echo time = 2.98 ms, flip angle = 9°, voxel size 1 mm × 1 mm × 1 mm). A gradient-echo echo-planar imaging (GE-EPI) sequence was used for imaging brain functional activity over 210 volumes or time points during resting state (40 axial slices, repetition time = 2400 ms, echo time = 25 ms, flip angle = 80°, voxel size 3.3 mm × 3.3 mm × 3.3 mm).

### Clinical and Neuropsychological Assessments

For the clinical characteristics, the disease stage was scored using the H&Y stage score, and the disease severity was captured by the Movement Disorder Society-Unified Parkinson’s Disease Rating Scale (MDS-UPDRS). Global cognition was assessed with the MoCA, because it is sensitive to the detection of executive deficits in PD (Zadikoff et al., 2008; Nazem et al., 2009) and consequently is a sensitive tool for the detection of PD dementia in the clinic than the traditional Mini-Mental State Examination (MMSE) (Hoops et al., 2009; Burdick et al., 2014). In addition, more details in cognitive domains were also assessed (Weintraub et al., 2015), including (i) memory [Hopkins Verbal Learning Test-Revised (HLVT-R)]; (ii) visuospatial function [Benton Judgment of Line Orientation (BJLO)] 15-item (split-half) version; (iii) processing speed-attention [Symbol-Digit Modalities Test (SDMT)]; and (iv) executive function and working memory [Letter-Number Sequencing (LNS) and semantic fluency (SF, animals, vegetables and fruits)]. Single scores were generated for each test; except for the HLVT-R, two scores were generated [i.e., immediate free recall (IFR) and delayed recognition hits (DRH)]. According to the diagnosis criteria of PD-MCI level I (Litvan et al., 2012), cognitive impairment was defined by two ways: (i) a recommended MoCA cut-off for PD of <26 was used (Dalrymple-Alford et al., 2010); (ii) using the detailed cognitive battery, cognitive impairment was defined as at least two test scores >1.5 standard deviations below the standardized mean score to support a PD-MCI diagnosis (Litvan et al., 2012). Clinical characteristics and neuropsychological assessments were presented in **Table 1**.

**Table 1:** Demographics of PD-NC, PD-MCI and HC groups.

	PD-MCI (*n* = 10)	PD-NC (*n* = 19)	HC (*n* = 13)	*p*
Gender (M:F)	5:5	14:5	11:2	0.182^a^
Age (years)	64.7 ± 7.0 (55.4–72.8)	59.1 ± 12.3 (38.2–77.2)	62.9 ± 9.0 (44–78.8)	0.332^a^
Disease duration (months)	5.4 ± 7.9 (1–25)	9.5 ± 10.8 (1–32)	–	0.304^b^
UPDRS total score	29.3 ± 9.6 (12–42)	25.7 ± 11.8 (10–53)	–	0.411^b^
H&Y stage	1.5 ± 0.5 (1–2)	1.3 ± 0.5 (1–2)	–	0.349^b^
MoCA	24.6 ± 2.4 (21–29)	28.4 ± 1.3 (26–30)	27.9 ± 1.1 (27–30)	<0.0001^a^
BJLO	11.7 ± 2.1 (8–14)	13.5 ± 1.8 (8–15)	12.8 ± 1.7 (10–15)	0.0487^a^
HVLT-R-DRH	11.2 ± 1.0 (9–12)	11.7 ± 0.5(11–12)	11.4 ± 1.1 (8–12)	0.328^a^
HVLT-R-IFR	22.6 ± 5.7 (13–32)	26.9 ± 4.5(18–35)	26.3 ± 4.4 (19–33)	0.0687^a^
SF total score	46.7 ± 11.3 (25–66)	47.6 ± 8.2 (33–63)	48.2 ± 9.5 (36–63)	0.928^a^
SDMT	35.8 ± 10.2 (20–49)	43.7 ± 7.6 (32–60)	47.5 ± 10.1 (28–65)	0.0133^a^

*^a^Comparisons were made among the three groups of PD-MCI, PD-NC and HC.*

*^b^Comparisons were made between PD-MCI and PD-NC groups.PD, Parkinson’s disease; PD-NC, Parkinson’s disease with normal cognition; PD-MCI, Parkinson’s disease with mild cognitive impairment; HC, healthy control. UPDRS, Unified Parkinson’s Disease rating scale; H&Y, Hoehn and Yahr; MoCA, Montreal cognitive assessment; BJLO, Benton judgment of line orientation; HVLT-R-DRH, delayed recognition hits in Hopkins verbal learning test-revised; HVLT-R-IFR, immediate free recall in Hopkins verbal learning test-revised; SF, semantic fluency; SDMT, symbol digit modalities test.*

### Data Preprocessing

Data preprocessing was carried out using Statistical Parametric Mapping (SPM12)^2^ and Data Processing Assistant for R-fMRI (DPARSF)^3^ toolkit (Yan and Zang, 2010). For MRI signal equilibrium and the participants’ adaptation to the scanning circumstance, the first 10 volumes of the functional images were discarded. The remaining 200 volumes were then corrected for intra-volume acquisition time delay between slices and for inter-volume geometrical displacement due to head motion. No participant was excluded under a criterion of the head displacement of >2 mm or angular rotation of >2° in any direction. Next, the individual T1-weighted images were co-registered to the mean realigned functional images using a linear transformation (Collignon et al., 1995), then were segmented into gray matter (GM), white matter (WM), and cerebrospinal fluid (CSF) tissue maps using a unified segmentation algorithm (Ashburner and Friston, 2005), and finally non-linearly normalized into the Montreal Neurological Institute (MNI) space using DARTEL approach (Ashburner, 2007). With the transformation parameters, all corrected functional volumes were spatially normalized into the MNI space, resampled to 3-mm isotropic voxels and spatially smoothed with a 4 mm full width at half maximum Gaussian kernel. Then, nuisance signals of 24 head motion parameters (Friston et al., 1995), global signal, CSF, and WM time series as well as linear trend were regressed out from each voxel’s time course. Finally, temporal band-pass filtering (0.01-0.1 Hz) was performed on the residual time series to reduce the effect of low-frequency drifts and high-frequency noise (Biswal et al., 1995; Lowe et al., 1998).

The ALFF calculation was performed using the DPARSF toolkit within a group GM mask that was generated by greater than 0.2 of the mean GM probability map of all 42 participants. As described in previous studies (Yang et al., 2007; Zang et al., 2007), the ALFF of each voxel is defined by averaged square root of the power spectrum of time series, computed in a frequency domain based on a fast Fourier transformation and averaged across the 0.01–0.1 Hz frequency interval. It was further divided by the global mean value to reduce the global effects of variability across participants.

### Statistical Analysis

To examine between-group differences in ALFF, a one-way analysis of covariance (ANCOVA) was first performed in a voxel-wise manner (within the group GM mask) under non-parametric permutation tests in a general linear model (GLM) among the three groups, with age, gender and data center treated as additional covariant factors. Here, we used Statistical non-Parametric Mapping software (SnPM13)^4^ to determine the significant voxels, and it provides an extensible framework for non-parametric permutation tests based on a GLM. The ANCOVA statistical significance level was not corrected by multiple comparisons but to just obtain a small volume mask using a threshold of *p* < 0.05 and cluster size >50 voxels, which could as much as possibly contain candidate voxels that may show truly significant differences between groups. The following subsequent *post hoc* analysis was performed within the mask, and the multiple comparisons were used with small volume correction based on non-parametric permutation tests, at a cluster-level threshold of *p* < 0.05 (*n* = 10000 permutations, FWE-corrected) with a cluster-forming threshold at the voxel level *p* < 0.01.

To investigate the relationship between regional ALFF values and clinical scores (i.e., UPDRS, MoCA) as well as cognitive domains (i.e., HLVT-R, BJLO, SDMT, and SF), we performed multiple linear regression analysis with age, gender and data center treated as additional covariates. First, we examined correlations of regional ALFF values within the regions that showed significant differences between the PD-MCI and HC with the UPDRS total score and the MoCA score in the PD-MCI group. Second, to examine possible contributions of cognitive domains in the relationships between the regional ALFF values and cognitive impairment, we examined correlations of regional ALFF values within the regions that showed significant differences between the PD-MCI and PD-NC with the test scores of cognitive domains (i.e., HLVT-R, BJLO, SDMT, and SF) in the PD-MCI group.

## Results

### Clinical and Demographic Testing of Sample

Clinical and demographic profiles of the 42 participants are shown in **Table 1**. There was no significant difference among the three groups in gender, age, DRH and IFR in HVLT-R (HVLT-R-DRH and HVLT-R-IFR) and SF scores. Moreover, no significant difference in disease duration, UPDRS total score and H&Y stage score was found between the PD-MCI and PD-NC subtypes. However, the PD-MCI patients had significantly lower MoCA, BJLO, and SDMT scores than the PD-NC and HC group, in consistent with clinical diagnosis of each subtype.

### Comparisons of ALFF Values Between Groups

**FIGURE 1:**
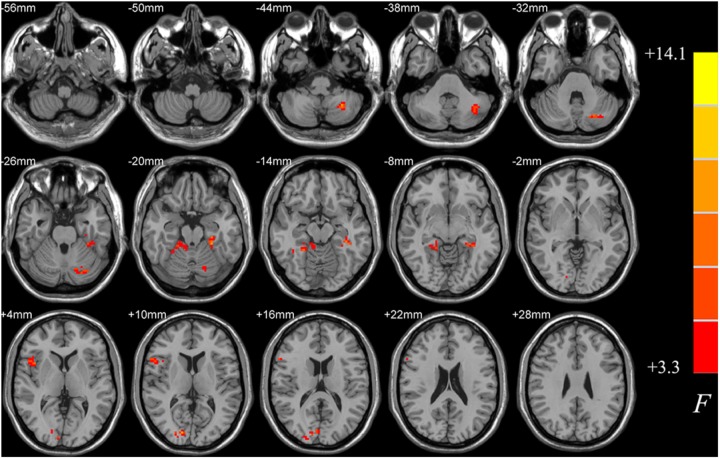
Differences in ALFF among PD-MCI, PD-NC, and HC groups. The statistical significance level of one-way analysis of covariance (ANCOVA, age, gender and data center treated as covariant factors) was not corrected by multiple comparisons but to obtain a small volume mask at a threshold of *p* < 0.05 and cluster size *k* > 50 voxels, which could as much as possibly contain candidate voxels showing truly significant differences between groups for the following *post hoc* analysis. ALFF, amplitude of low-frequency fluctuations; PD-MCI, Parkinson’s disease with mild cognitive impairment; PD-NC, Parkinson’s disease with normal cognition; HC, healthy control.

ANCOVA analysis revealed a range of regions showing uncorrected significant differences in ALFF among the PD-MCI, PD-NC, and HC groups (*p* < 0.05, cluster size *k* > 50, uncorrected; see **Figure 1**). These areas mainly covered regions of inferior cerebellum, occipital, right fusiform and right inferior frontal lobes. This analysis aimed to result as a small volume mask for the following *post hoc* analysis, which could as much as possibly contain candidate voxels that may show truly significant differences between PD-MCI and NC, between PD-NC and HC, or between PD-MCI and PD-NC.

**FIGURE 2:**
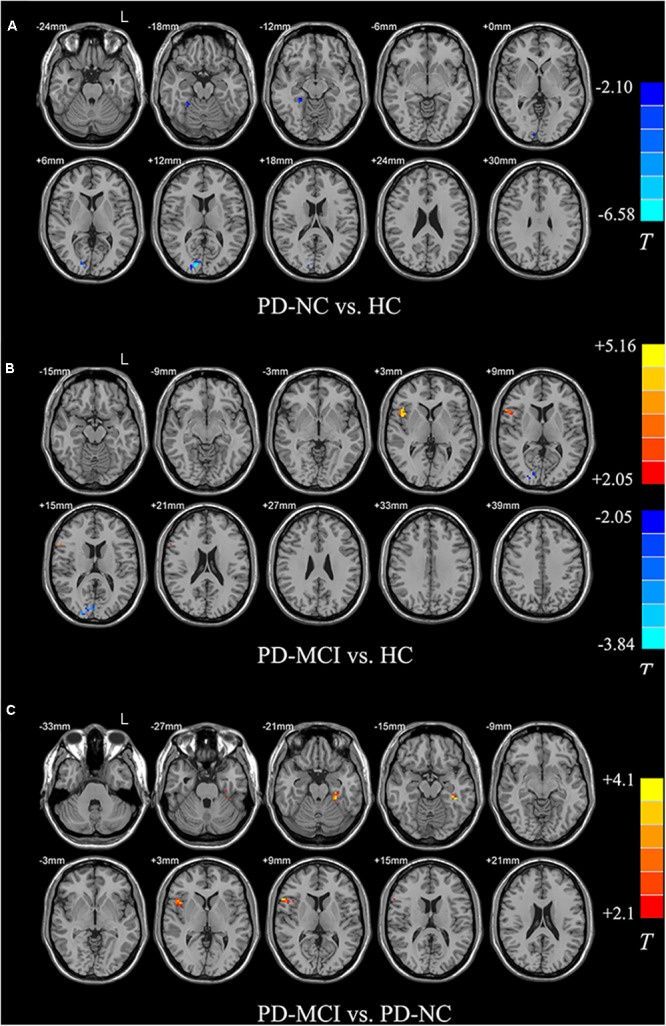
Differences in ALFF between groups. All the multiple comparisons were performed with a small volume correction based on non-parametric permutation tests, at a cluster-level threshold of *p* < 0.05 (*n* = 10000 permutations, FWE-corrected) with a cluster-forming threshold at the voxel level *p* < 0.01. **(A)** Differences in ALFF between PD-NC and HC; **(B)** Differences in ALFF between PD-MCI and HC; **(C)** Differences in ALFF between PD-MCI and PD-NC. For details of the significant regions, see **Table 2**.

Compared with the HC, both PD groups exhibited ALFF decreases in the occipital areas (i.e., Calcarine_R/Cuneus_R), and the PD-NC group additionally showed ALFF decreased in the right fusiform area as well (*p* < 0.05; see **Figures 2A,B** and **Table 2**). Specially, the PD-MCI group additionally exhibited increased ALFF in the opercular part of right inferior frontal lobe (i.e., Frontal_Inf_Oper_R) (*p* < 0.05; see **Figure 2B** and **Table 2**). Comparing the PD-NC, the PD-MCI group exhibited significantly higher ALFF in the Frontal_Inf_Oper_R and left fusiform gyrus (*p* < 0.05; see **Figure 2C** and **Table 2**).

**Table 2:** Between-group ALFF differences of regional brain activity.

Brain regions (AAL)	Brodmann area	MNI coordinates (mm)			*T* value	Cluster size
		*X*	*y*	*z*		
**PD-MCI vs. HC**						
Frontal_Inf_Oper_R	BA 44/45	42	12	3	5.16	54
Calcarine_R/Cuneus_R	BA 18	12	-84	12	-3.84	30
**PD-NC vs. HC**						
Calcarine_R/Cuneus_R	BA 18/17	15	-90	12	-6.58	39
Fusiform_R	BA 37	27	-42	-15	-3.61	20
**PD-MCI vs. PD-NC**						
Frontal_Inf_Oper_R	BA 45	57	18	6	4.10	45
Fusiform_L	BA 36	-27	-36	-21	4.13	34

*PD, Parkinson’s disease; PD-NC, Parkinson’s disease with normal cognition; PD-MCI, Parkinson’s disease with mild cognitive impairment; HC, healthy control; BA, Brodmann area; R, right; L, left. All the coordinates are denoted by MNI space coordinates.*

### Correlation of ALFF Values With Clinical and Neuropsychological Assessment in the PD-MCI Group

**FIGURE 3:**
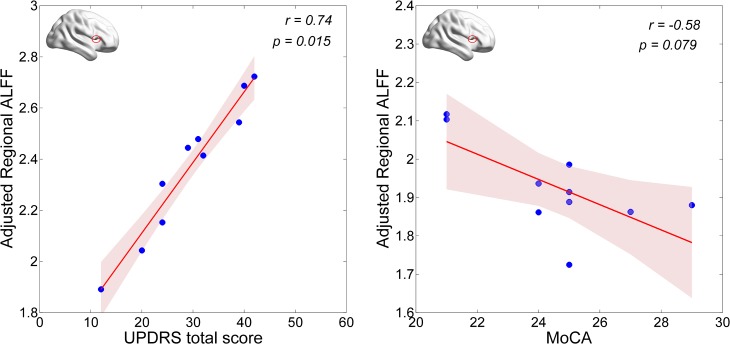
Correlations of regional ALFF values with the UPDRS total score and MoCA score.

**FIGURE 4:**
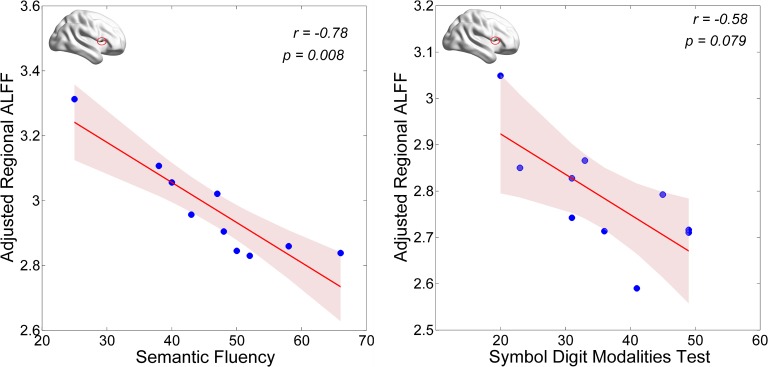
Correlations of regional ALFF values with the scores of cognitive domains.

First, we examined correlations of regional (i.e., Calcarine_R/Cuneus_R and Frontal_Inf_Oper_R) ALFF values with the UPDRS total score and the MoCA score in the PD-MCI group. We found that the neural activity in the Frontal_Inf_Oper_R area was positively correlated with the UPDRS total score (*p* < 0.05), but marginally negatively correlated with the MoCA score (see **Figure 3**). Second, we also examined correlations of regional (i.e., Frontal_Inf_Oper_R and left fusiform gyrus) ALFF values with the test scores of cognitive domains. Interestingly, the neural activity in the Frontal_Inf_Oper_R showed a significantly negative correlation with the score of SF test (the executive function and working memory) (*p* < 0.01) and a marginally negative correlation with the score of SDMT (the processing speed-attention) (see **Figure 4**).

## Discussion

Cognitive dysfunction occurs frequently in patients with PD. They are highly relevant, and limit the patients’ quality of life and increase caregiver burden (Leroi et al., 2012). A range of 18.9–38.2% of PD patients are diagnosed as PD-MCI across studies as a risk factor or a harbinger of subsequent dementia (Litvan et al., 2011), and about 30% of PD patients develop to PD dementia (Aarsland and Kurz, 2010). Moreover, common features such as visual hallucinations, depression, fluctuating cognition, parasomnias, autonomous disturbance, and apathy in PD are also associated with cognitive impairment. Thus, investigation of abnormality of brain activity related to mild cognitive impairment in PD is very important not only for the PD treatment and management but also the further understanding of neuronal and pathophysiological mechanisms in PD development.

Recent R-fMRI studies have indicated that the ALFF is physiologically meaningful for measuring intrinsic or spontaneous neuronal activity of the brain (Biswal et al., 1995; Zang et al., 2007; Wang et al., 2011). By using the ALFF measurement, our study revealed that both PD-MCI and PD-NC groups displayed hypoactivity (i.e., reduced spontaneous brain activity) in the occipital cortex (i.e., Calcarine_R/Cuneus_R) compared with the HC group. This finding was also reported in a previous study using a dataset of 58 patients with early to moderate stage PD and 54 healthy controls (Wu et al., 2015) and a meta-analysis (Pan et al., 2017). More emerging evidence has indicated crucial involvement of the occipital lobe in the pathophysiology of PD. Morphological analysis indicated reduction in cortical thickness in the occipital lobe (Baggio et al., 2015). Using graph-theoretical analysis, Fang et al. (2017) found that in the early disease stage, patients with PD displayed significant lower network efficiency in the bilateral occipital lobe (Fang et al., 2017). Furthermore, a recent PET study indicated that both the cognitively normal PD and the PD-MCI groups had reduced FDG metabolism in the occipital lobe, as well as in the inferior parietal and posterior temporal regions in the PD-MCI group. To a certain degree, the reason may be due to a crucial rule in visual-spatial processing in the occipital lobe, which should be related with hallucination (Yao et al., 2015) and other common dysfunction in PD. Moreover, a longitudinal study over 2 years using H2(15)O PET has observed decreased learning-related activation in parietal and temporal-occipital association areas at the second session in PD (Carbon et al., 2010). Therefore, we postulate that reduction in occipital activity is associated with the PD development.

The cognitive profile in PD-MCI is heterogeneous, but differs from that of MCI due to AD by showing relatively more severe visuospatial and executive deficits and relatively less severe memory impairment (Aarsland, 2016). In the present study, we found lower level of visuospatial (i.e., measured by BJLO) and processing speed-attention functions (i.e., measured by SDMT) in the PD-MCI, which is consistent with the characteristics of cognitive impairment in PD-MCI. Moreover, hyperactivity in the opercular part of right inferior frontal lobe (i.e., Frontal_Inf_Oper_R) was observed in the PD-MCI, compared to both HC and PD-NC groups. This region has been broadly suggested to play an important role in executive control function in many task-fMRI studies (Hampshire et al., 2010). Hyperactivity in the region could be explained by greatly improving its functional performance in favor of compensating a certain degree of cognitive decline and preserving the global cognition in early PD with MCI. The ALFF-clinical correlation results may support this presumption. The regional ALFF in this region was marginally negatively correlated with general cognition as measured by MoCA in the PD-MCI (see **Figure 3**). With regarding to cognitive domains, the present study showed a significantly negative correlation with the score of SF test (the executive function and working memory) and a marginally negative correlation with the score of SDMT (the processing speed-attention) (see **Figure 4**). Thus, these findings may indicate that more cognitive deficit induced higher neural activity in the right inferior frontal lobe. In a cognitive task study (planning set-shift task), Nagano-Saito et al. (2016) found a similar phenomenon, that was increased activation in the frontal and other related areas during the planning the set-shift task at the second session when the behavior performance had inclined to be decreased in the PD-MCI patients. So, we suggested that hyperactivity in the opercular part of right inferior frontal lobe may reflect an adaptive compensatory effect in response to a modest cognitive decline in early PD with MCI.

Moreover, the presence of MCI at early stages of PD could affect both cognitive and motor corticostriatal loops (Nagano-Saito et al., 2014). The spontaneous neural activity in the right inferior frontal gyrus was additionally positively correlated with disease severity as measured by UPDRS total score, which may indicate the region as an indicator of disease progression of total PD symptoms as well. Nevertheless, whether and how the right inferior frontal gyrus contributes or interacts with cognitive function in PD remain to be studied in the future.

In addition, we also found hyperactivity in the left fusiform in the PD-MCI. Similar findings have been found in AD or MCI patients during both cognitive task and resting state. For example, Wang et al. (2011) have showed increased activity in ALFF in both AD and MCI patients compared to healthy controls, and so does the functional homogeneity of spontaneous brain activity in AD patients (He et al., 2007). Prvulovic et al. (2002) observed that the fusiform increased functional activation in AD patients who were performing a visuospatial processing task. Because the fusiform spatially locates close to the parahippocampal gyrus, it may serve as a role of compensation for abnormality of the parahippocampal gyrus associated memory deficit in PD-MCI. Indeed, the region is mainly involved in the processing of memory (Kohler et al., 1998) except for facial processing. However, it is unclear about the differences of compensatory mechanism in the fusiform between the MCI due to AD and MCI in early PD.

## Limitations

We acknowledge several limitations in our study. First, the sample size in the present study was relatively small. Because the PPMI project mainly focused on structural MRI and diffusion tensor imaging (DTI), very few R-fMRI scanning has been conducted. Second, other MRI modality and gene data were not investigated in this study. The underlying basis of structural (cortical Morphological) and functional connectivity or network and genetics of impaired brain activity in the occipital lobe and hyperactivity in the inferior frontal gyrus needs to be investigated in the future. Recently, several studies have found a range of abnormal functional network that is associated with cognitive decline in PD. For example, a white-matter (WM) study has revealed that PD-MCI patients showed a distributed pattern of WM abnormalities, including anterior and superior corona radiata, genu, and body of the corpus callosum, and anterior inferior fronto-occipital, uncinate, and superior longitudinal fasciculi (Agosta et al., 2014). R-fMRI functional connectivity analysis suggested a wide range of connectivity dysfunction in the default mode network, dorsal attentional network and frontoparietal networks (Baggio et al., 2015; Hou et al., 2016).

## Conclusion

In summary, our study concluded hyperactivity (i.e., reflect a compensatory recruitment) in the opercular part of right inferior frontal gyrus and hypoactivity in the occipital areas in early PD with MCI, which may be associated with cognitive decline and contribute to thought of PD dementia progression and further clinical treatment in PD. Further investigations of longitudinal changes and interaction in brain activity with the right inferior frontal gyrus as well as intervention are needed in future.

## Author Contributions

ZW conceived and analyzed the study, and wrote the manuscript. XJ arranged the clinical data and participated in the manuscript revision. HC and TF were involved in the writing and discussion. HW contributed to clinical diagnosis and was involved in writing and discussion. All authors read and approved the final manuscript.

## Conflict of Interest Statement

The authors declare that the research was conducted in the absence of any commercial or financial relationships that could be construed as a potential conflict of interest. The reviewer CL declared a shared affiliation, with no collaboration, with several of the authors, XJ, HC, and TF to thehandling Editor.

## Footnotes

1 http://www.ppmi-info.org/

2 http://www.fil.ion.ucl.ac.uk/spm

3 http://www.restfmri.net/forum/DPARSF

4 http://warwick.ac.uk/snpm
